# Uterine smooth muscle tumors of uncertain malignant potential (STUMP): A case report

**DOI:** 10.1016/j.ijscr.2024.109607

**Published:** 2024-04-04

**Authors:** Fatima El Hassouni, Samia Tligui, Asmaa Lakhdissi, Lamiaa Rouas, Mounia El Youssfi

**Affiliations:** a Department of Obstetrics and Gynecology, Oncology and High-Risk Pregnancies, Maternity Hospital Souissi, Ibn Sina university Hospital, Rabat, Morocco; b Mohamed V University, Rabat, Morocco; c Department of Medical Oncology, National Institute of Oncology, Rabat, Morocco; d Department of Pathology, Ibn Sina university Hospital, Rabat, Morocco

**Keywords:** Uterine smooth muscle tumor of uncertain malignant potential, Leiomyosarcoma, STUMP, Atypical leiomyoma.

## Abstract

**Introduction:**

Uterine smooth muscle tumors of uncertain malignant potential (STUMP) are extremely rare, defined as a uterine smooth muscle tumors that cannot be diagnosed as benign or malignant and does not satisfy all the criteria for leiomyosarcoma or leiomyoma.

**Case representation:**

A 48-year-old woman who presented with a history of heavy menstrual bleeding and pelvic pain. Physical examination revealed an enlarged uterus. Ultrasonography showed lobular and enlarged uterus containing multiples leiomyomas. A subtotal hysterectomy was performed. A Pathological analysis revealed a uterine mass diagnosed as a smooth muscle tumor of uncertain malignant.

**Discussion:**

Uterine STUMPs are rare and are commonly diagnosed by histopathological evaluation following myomectomy or hysterectomy. The most common clinical manifestations of uterine STUMP are the same as leiomyomas. Prognosis for the patient is unclear and there is a risk of recurrence with the tumors. However, considering their potential risk of recurrence and metastasis, it is advisable to maintain six-monthly controls for 5 years and then annual controls for 5 years more.

**Conclusion:**

Due to the rarity of uterine STUMP, there are no specific guidelines for their treatment and control. The scientific literature needs to be constantly updated in order to identify masses suspected of malignancy before surgery and improve patient management and follow-up.

## Highlights

• Uterine smooth muscle of uncertain malignant potential (STUMP) are extremely rare
• Uterine STUMPs are commonly diagnosed by histopatholological evaluation following myomectomy or hysterectomy.
• Total hysterectomy is an optimal treatment for stumps among women without a desire of fertility to prevent recurrences.
• All uterine STUMP cases should be the object of a multidisciplinary approach

## 1 Introduction

Uterine smooth muscle tumors encompass a diverse group of neoplasms. Among them, myomas stand out as the most common benign gynaecological tumors, while leiomyosarcomas account for 60–70 % of uterine sarcomas [1]. Uterine smooth muscle tumor of uncertain malignant potential (STUMP) are extremely rare, defined by The World Health Organization (WHO) as a uterine smooth muscle tumors that cannot be diagnosed as benign or malignant and does not satisfy all the criteria for leiomyosarcoma [2]. These criteria are based on degrees of cellular atypia, mitotic counts, and tumor cell necrosis [3].

The diagnosis of STUMP is made following myomectomy or hysterectomy and represents 0.01 % of these procedures [4]. As these tumors persist in presenting clinical challenges, this report aims to contribute to a better understanding of their clinical presentation, management strategies, and outcomes.

We report a case of uterus STUMP diagnosed in a patient with a history of myomectomy and analyse the clinical–pathological characteristics, treatment, and follow-up of STUMPs.

## 2 Case report

A 48-year-old multiparous woman was admitted to our hospital's gynecology ward with a history of heavy menstrual bleeding and pelvic pain. She had undergone an abdominal myomectomy in 2006, a caesarean section in 2010 and hypertension under treatment. Physical examination revealed an enlarged uterus. Subsequent pelvic ultrasound showed markedly lobular and enlarged uterus containing intramural uterine leiomyomas, the two largest measuring respectively 6.4 × 5.4 cm, and 4.2 × 4.8 cm. Blood testing found a haemoglobin level of 7.4 g/dl although the patient's vital signs were stable.

After discussing the various treatment options, the patient consented to hysterectomy by laparotomy. Intraoperative examination showed severe adhesions fusing the bladder to the uterus and the uterus to the small bowel. After adhesiolysis, intra-abdominal visualization revealed an enlarged uterus containing two fundal intramural myomas as diagnosed by ultrasound. The adnexa on both sides were normal and there were no fluid collections or other significant findings suggestive of malignancy throughout the abdomen. Due to the many difficulties associated with the total hysterectomy, including obesity, anaemia, a large uterus and adhesions from previous operations, a subtotal hysterectomy was decided in order to complete the procedure safely. The patient was discharged a few days after surgery without any complications.

**Fig. 1:**
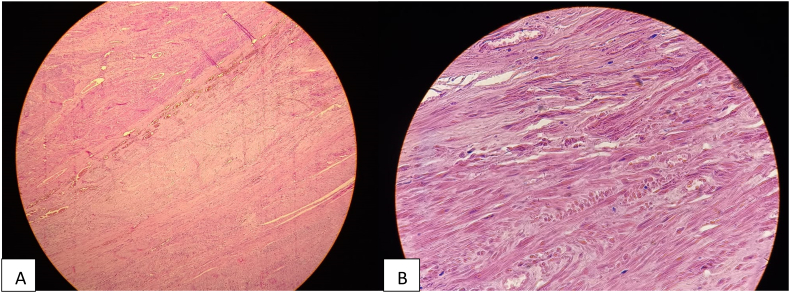
Low power view with a proliferation of spindle smooth muscle cells at the lower right corner and a large eosinophilic zone of tumor cell necrosis in the upper left corner. HE X 20. STUMP. Coagulative tumor cell necrosis with the ghost of spindle smooth muscle cells. HE X 40.

**Fig. 2:**
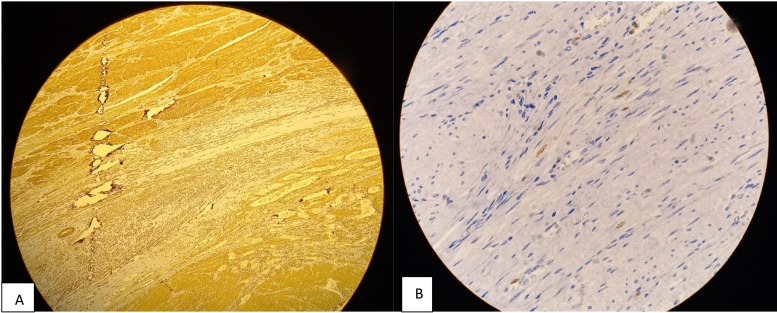
Stump. Spindle cells. Positive stain with smooth muscle actin in the viable zones at the upper left corner and the coagulative tumor cell necrosis in the lower right corner. Stump with spindle cell morphology and low mitotic activity with few cigar shaped nuclei positif with Ki67. Immunohistochemistry.

**Fig. 3:**
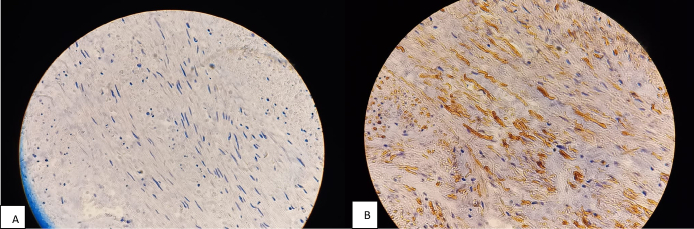
STUMP with fusiform cells. p 53 wild type. STUMP. Smooth muscle cells are desmin-positive.

Macroscopic examination revealed a bosselated, large uterus with multiple leiomyomas. Microscopic examination showed tumors with features of conventional leiomyoma and a mesenchymal tumor proliferation with a fasciculate architecture composed of fusiform tumorous cells with STUMP. It showed cell necrosis with atypical cells and karyorrhexis; however, there was no evidence of a mitotic activity (Fig. 1). On immunohistochemical analysis, the smooth muscle cells expressed AML (Fig. 2, A), P53 wild-type (Fig. 3, A) and desmin (Fig. 3, B). The Ki-67 proliferation index was low (<1 %) (Fig. 2, B).

For optimal oncological care, our case was discussed in a multidisciplinary team meeting (MDTM). The consensus was that no adjuvant therapy would be offered considering the lack of benefit of adjuvant therapy in the current literature. However, it was recommended to keep the patient in close follow-up, every six months for five years, by a gynaecologic oncologist, including clinical and computed tomography (CT) scan. A 6-month follow-up thoraco-abdomino-pelvic CT scan revealed no evidence of local recurrence or distant metastasis.

## 3 Discussion

Mesenchymal smooth muscle cell tumors constitute the predominant manifestation of uterine neoplasia, enveloping leiomyomas, mesenchymal STUMP, and leiomyosarcomas [3]. Due to the absence of currently reported specific symptoms and preoperative diagnostic methods, STUMP is typically diagnosed postoperatively based on histological findings [3]. Research and large observational studies are needed in patients with STUMP undergoing surgery to identify masses suspected of malignancy before surgery.

In the study conducted by Borella and al., the average age at the time of diagnosis was 46 years [5] which is similar to other studies [6]. Additionally; other research indicates that a younger age at the time of diagnosis is correlated with an increased likelihood of disease recurrence [7].

STUMP share similar clinical features with leiomyomas with either benign or malignant conditions, which include abnormal uterine bleeding, pelvic pain or pressure and anaemia [4,6].

Ultrasonography is the first-line examination employed as a diagnostic tool for uterine tumors in routine. However, studies have indicated its limited effectiveness in distinguishing stumps from leiomyoma or leiomyosarcoma [3]. Stumps usually appear as large, oval-shaped tumors with internal heterogeneous echogenicity on ultrasound and circumferential or intralesional vascularization on Doppler imaging [6]. Other investigations suggest that a solitary irregular tumor, lacking acoustic shadowing but displaying the presence of free fluid, is closely linked to STUMP. A recent study by Russo and al. Highlighted the potential of patient age, tumor size, and intralesional/circumferential vascularity to aid in tumor differentiation [8].

On MRI, STUMP typically displays a higher T2 signal intensity compared to leiomyomas and demonstrates heterogeneous post-contrast enhancement. High-intensity signals on Diffusion-Weighted Imaging (DWI) and low Apparent Diffusion Coefficient (ADC) values are associated with leiomyosarcomas or stumps [3].

Recent study made by Ho et al. Recently reported significantly elevated maximal standardized uptake values and metabolic tumor-to-necrosis ratios in leiomyosarcomas (LMS) and stumps compared to leiomyomas in positron emission tomography (PET). Moreover, all LMS and STUMP cases showed a distinct “hollow ball” sign on fluorodeoxyglucose (FDG) uptake, corresponding to regions of coagulative tumor necrosis, a feature absent in patients with leiomyomas [9].

Pathologically, the diagnosis of STUMP requires at least one of these three diagnostic indicators: mitotic count, cell atypia, and tumor cell necrosis, with one of them being required. Currently, the diagnosis is based on criteria established by the World Health Organization (WHO) in 2014:

1. Focal or multifocal moderate-to-severe cell atypia, mitotic count <10/10 high-power field (HPF), and undefined necrosis.
2. Diffuse moderate-to-severe cell atypia, mitotic count <10/10 HPF, and no tumor necrosis.
3. Tumor necrosis, with mild or absent cell atypia and a mitotic count <10/10 HPF.
4. Mitotic count >15/10 HPF and no tumor necrosis or cell pleomorphism. [2]

The expression of immunohistochemical markers (p53, p16) could be helpful in the prognostic stratification of STUMP [10]. Study findings have indicated that pathological characteristics, epithelioid features, high proliferation activity, low progesterone receptor expression, and diffuse p16 expression are associated with disease recurrence and shorter recurrence-free survival [5].

Total hysterectomy is an optimal treatment for stumps compared with myomectomy among women without a desire of fertility to prevent recurrences [3]. Currently, there is no consensus regarding the use of adjuvant hormone therapy or chemotherapy but it is recommended to keep the patient in close follow-up [4]. For patients who have undergone hysterectomy, follow-up assessments are suggested every six months for the initial five years, followed by yearly evaluations thereafter [3]. These check-ups should encompass gynaecologic examinations, chest radiography, abdominopelvic ultrasound, and an annual whole abdomen computed tomography (CT) scan [9]. In cases where myomectomy was performed for fertility preservation, an annual pelvic MRI is considered a suitable alternative to the CT scan [4,9].

## 4 Conclusion

Uterine STUMP is a rare tumor which is generally diagnosed at the pathologic examination. The preoperative diagnosis is quite impossible, and tumor is detected at the definitive pathologic examination. STUMP is a challenge for both the physicians and patients and further studies are crucial to identify diagnostic tests that aid in the early suspicion of these tumors before surgery. Considering STUMP as a potential differential diagnosis will enhance patient management and facilitate treatment and follow up, leading to a greater consensus.

Our work has been reported in line with the SCARE Guidelines 2020 criteria [11].

## Consent

Written informed consent was obtained from the patient to publish this case report and accompanying images. On request, a copy of the written consent is available for review by the Editor-in-Chief of this journal.

## Ethical approval

Ethical approval is not applicable. The case report is not containing any personal information.

## Funding

No funding or grant support.

## CRediT authorship contribution statement

Fatima Elhassouni, Mounia El Yousfi: performed surgery, paper writing and editing.

Mounia El Yousfi, Lamiaa Rouas: literature review, Supervision.

Fatima Elhassouni, Samia Tligui, Lamiaa Rouas: Manuscript editing, picture editing.

## Guarantor

Fatima Elhassouni M.D., Department of Obstetrics and Gynecology, Oncology and High-Risk Pregnancies, Maternity Souissi, University Hospital Center IBN SINA, University Mohammed V, Rabat, Morocco.

Email: fatimaelhassouni@hotmail.fr.

## Research registration number

Not applicable.

## Declaration of competing interest

The authors declare that they have no competing interests relevant to the content of this article.
